# Magnetic properties of (Bi_1−x_La_x_)(Fe,Co)O_3_ films fabricated by a pulsed DC reactive sputtering and demonstration of magnetization reversal by electric field

**DOI:** 10.1038/s41598-021-90547-2

**Published:** 2021-05-27

**Authors:** Munusamy Kuppan, Daichi Yamamoto, Genta Egawa, Sivaperuman Kalainathan, Satoru Yoshimura

**Affiliations:** 1 Center for Regional Revitalization in Research and Education , Akita University, Akita, 010-8502 Japan; 2 Graduate School of Engineering Science, Akita University, Akita, 010-8502 Japan; 3 Centre for Crystal Growth, Vellore Institute of Technology, Vellore, Tamil Nadu 632014 India; 4 Present Address: Materials Research Center, Technology & IP HQ, TDK Corporation, Ichikawa, 272-0026 Japan

**Keywords:** Materials science, Materials for devices, Electronic devices

## Abstract

(Bi_1−x_La_x_)(Fe,Co)O_3_ multiferroic magnetic film were fabricated using pulsed DC (direct current) sputtering technique and demonstrated magnetization reversal by applied electric field. The fabricated (Bi_0.41_La_0.59_)(Fe_0.75_Co_0.25_)O_3_ films exhibited hysteresis curves of both ferromagnetic and ferroelectric behavior. The saturated magnetization (*M*_s_) of the multiferroic film was about 70 emu/cm^3^. The squareness (*S*) (= remanent magnetization (*M*_r_)/*M*_s_) and coercivity (*H*_c_) of perpendicular to film plane are 0.64 and 4.2 kOe which are larger compared with films in parallel to film plane of 0.5 and 2.5 kOe. The electric and magnetic domain structures of the (Bi_0.41_La_0.59_)(Fe_0.75_Co_0.25_)O_3_ film analyzed by electric force microscopy (EFM) and magnetic force microscopy (MFM) were clearly induced with submicron scale by applying a local electric field. This magnetization reversal indicates the future realization of high performance magnetic device with low power consumption.

## Introduction

Magnetic reversal using an electric field is a promising and future technology for multifunctional devices due to its lower power consumption. Multiferroic materials with magneto-electric effect, which simultaneously exhibit spontaneous polarization and magnetization, have been receiving greater attention for this system. BiFeO_3_ is a multiferroic material with a high ferroelectric Curie temperature (*T*_C_) of 1120 K and a high antiferromagnetic Neel temperature (*T*_N_) of 640 K. However, generally say, BiFeO_3_ films have a high leakage current density at room temperature^1^, so several methods such as substitution of atoms had been performed for reduction of the leakage current density and improvement of ferroelectric property of BiFeO_3_ films. For example, substitution of Er^2^, La^3,4^, or Sc^5^) for Bi, and substitution of Cr^6,7^, Mn^8,9^, or Ti^10,11^ for Fe. To apply magnetic devices of these BiFeO_3_ based films, improvement of their ferromagnetic property such as large saturation magnetization (*M*_s_), large coercivity (*H*_c_), and perpendicular magnetic anisotropy is more important. The substitution of atoms is also effective for the improvement of their ferromagnetic property. Suitable multiferroic materials with ferromagnetism with high *M*_s_ and ferroelectricity at room temperature, such as (Bi_1−x_Ba_x_)FeO_3_ powder^12^ have been reported. Multiferroic films with ferromagnetism and ferroelectricity at room temperature, such as (Bi_1−x_La_x_)FeO_3_ films^13^ and Bi(Fe,Co)O_3_ films^14–16^ also have been reported. However, the magnetic properties of the films were not sufficient for device application. As mentioned above, large *M*_s_, large *H*_c_, and perpendicular magnetic anisotropy are important. In our previous study, we succeeded to fabricate the highly qualified (Bi_0.48_Ba_0.52_)FeO_3_ films with ferromagnetism (*M*_s_ : 90 emu/cm^3^) and ferroelectricity^17^ by using a pulsed DC (direct current) reactive sputtering technique^18^. The *M*_s_ of this film was 1.5 times larger than that of (Bi_0.46_Ba_0.54_)FeO_3_ films fabricated by using an normal RF direct sputtering method^19^ and was similar to that of (Bi_1−x_Ba_x_)FeO_3_ powder^12^, this means that the quality of the (Bi_1−x_Ba_x_)FeO_3_ films fabricated by pulsed DC reactive sputtering technique with suitable sputtering condition is high. And also we have succeeded to demonstrate the magnetization reversal by local electric field application^17^. However, the *H*_c_ and the perpendicular magnetic anisotropy of the films were not sufficient for high performance magnetic device application. To change the intrinsic magnetic property, investigation of substitution materials against Bi (A site) and Fe (B site) should be suitable. For BiFeO_3_-based films, substitution of A-site with La^13^, substitution of B-site with Co^14–16^ were reported for the introduction of ferromagnetism. Therefore, substitution with both materials will have large effectiveness for introduction of ferromagnetism. In this study, to improve the magnetic properties of BiFeO_3_-based films, we aimed to produce highly qualified La and Co doped BiFeO_3_ films by using reactive pulsed DC sputtering method.

## Results and discussion

**Figure 1:**
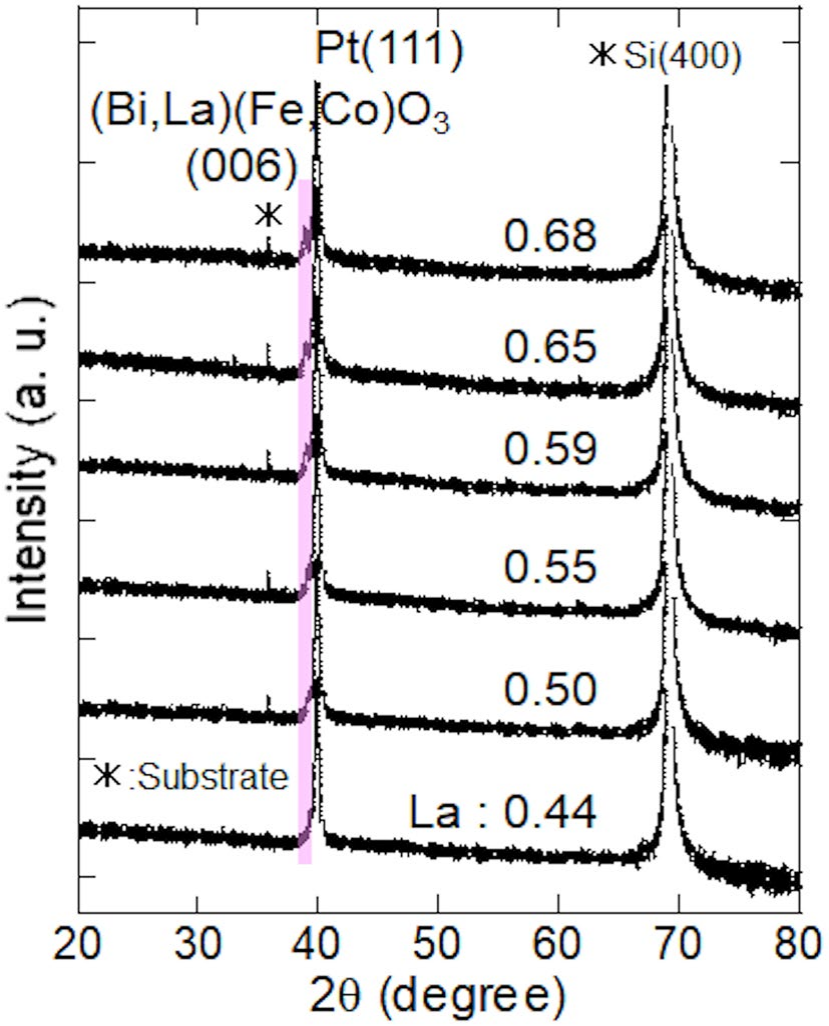
XRD profile of Bi–La–Fe–Co–O film with various La concentration on Ta/Pt layer fabricated by pulsed DC reactive sputtering.

Figure 1 shows the X‐ray diffraction (XRD) profile of Bi–La–Fe–Co–O film on Ta/Pt layer fabricated by pulsed DC reactive sputtering. From previous study, crystallization of Bi–Ba–Fe–O films on Ta/Pt layer fabricated by pulsed DC reactive sputtering was accelerated compared with the case of Bi–Ba–Fe–O films fabricated by RF (radio frequency) direct sputtering^17^. Therefore, this indicates that the pulsed DC reactive sputtering is useful for fabrication of oxide films with high quality. The Pt underlayer was found to have a strong (111) orientation. The Bi–La–Fe–Co–O films were found to have (006) peak; this indicates that these films have a (001) orientation. Here, the peak at around 36° is from the substrate. With increasing La concentration, the (006) peak shifts to a lower angle. Here, the similar peak shifts were observed in the case of Bi–Ba–Fe–O films with increasing Ba concentration^17,19^. This indicates that the Bi atoms of the Bi(Fe,Co)O_3_ phase are replaced by La atoms.

**Figure 2:**
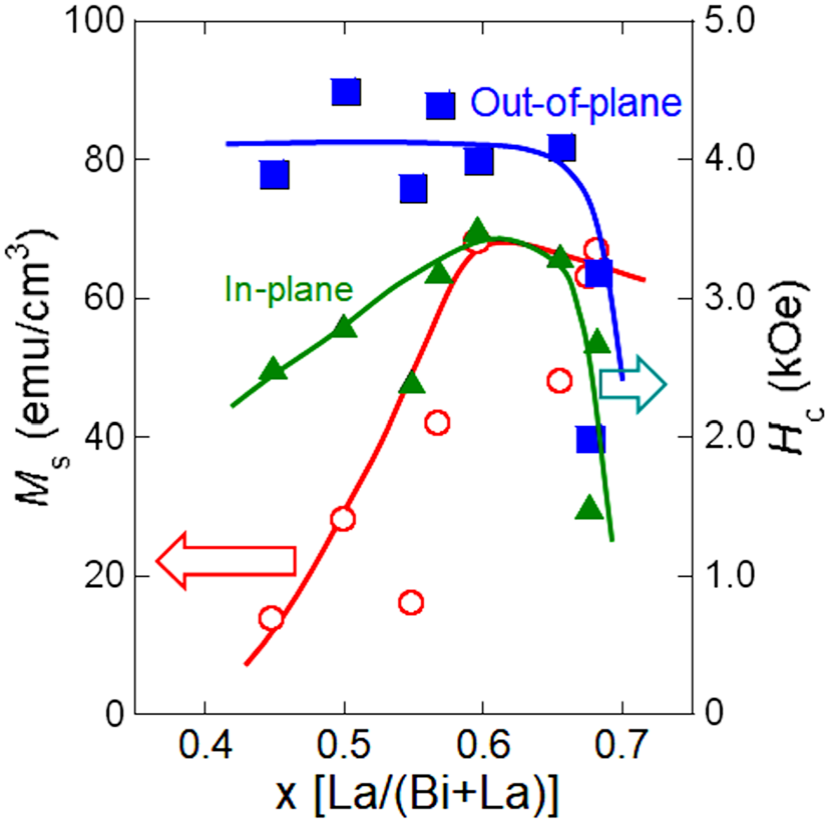
Dependence of saturation magnetization and coercivity on La concentration in Bi–La–Fe–Co–O films fabricated by pulsed DC reactive sputtering.

Figure 2 shows the dependence of the *M*_s_ and *H*_c_ (in-plane and out-of-plane) measured by vibrating sample magnetometer (VSM) with the magnetic field of parallel and perpendicular to film plane on La concentration in the Bi–La–Fe–Co–O films fabricated by pulsed DC reactive sputtering. As mentioned before, the *M*_s_ in the (Bi_0.48_Ba_0.52_)FeO_3_ film fabricated by pulsed DC reactive sputtering is 1.5 times larger than that in the (Bi_0.46_Ba_0.54_)FeO_3_ film fabricated by normal RF direct sputtering method^17,19^. The reason of the large *M*_s_ in the Bi–Ba–Fe–O films fabricated by pulsed DC reactive sputtering was improvement of crystalline structure. With increasing La concentration up to around 0.6, *M*_s_ of the Bi–La–Fe–Co–O films increased up to 70 emu/cm^3^. The reason of the large *M*_s_ in this Bi–La–Fe–Co–O films fabricated by pulsed DC reactive sputtering is also crystalline structure with high quality. The reason of low *M*_s_ in the Bi–La–Fe–Co–O films with the La concentration of around 0.4 is due to the residual of BiFeO_3_ antiferromagnetic phase. With increasing La concentration more than 0.6, the *M*_s_ and *H*_c_ decreased. In the case of Bi–Ba–Fe–O films with the Ba concentration of more than 0.60, the *M*_s_ and *H*_c_ also decreased. Here, in the case of Bi–Ba–Fe–O films with the Ba concentration of more than 0.70, clear other phase was observed from XRD analysis^17^. Therefore, one of the reasons of the decrease in *M*_s_ and *H*_c_ (in-plane and out-of-plane) in the Bi–La–Fe–Co–O films with the La concentration of more than 0.65 is due to the inhibition of the formation of the (Bi_1−x_La_x_)(Fe_0.75_Co_0.25_)O_3_ ferrimagnetic phase and generation of other phase with paramagnetism.

**Figure 3:**
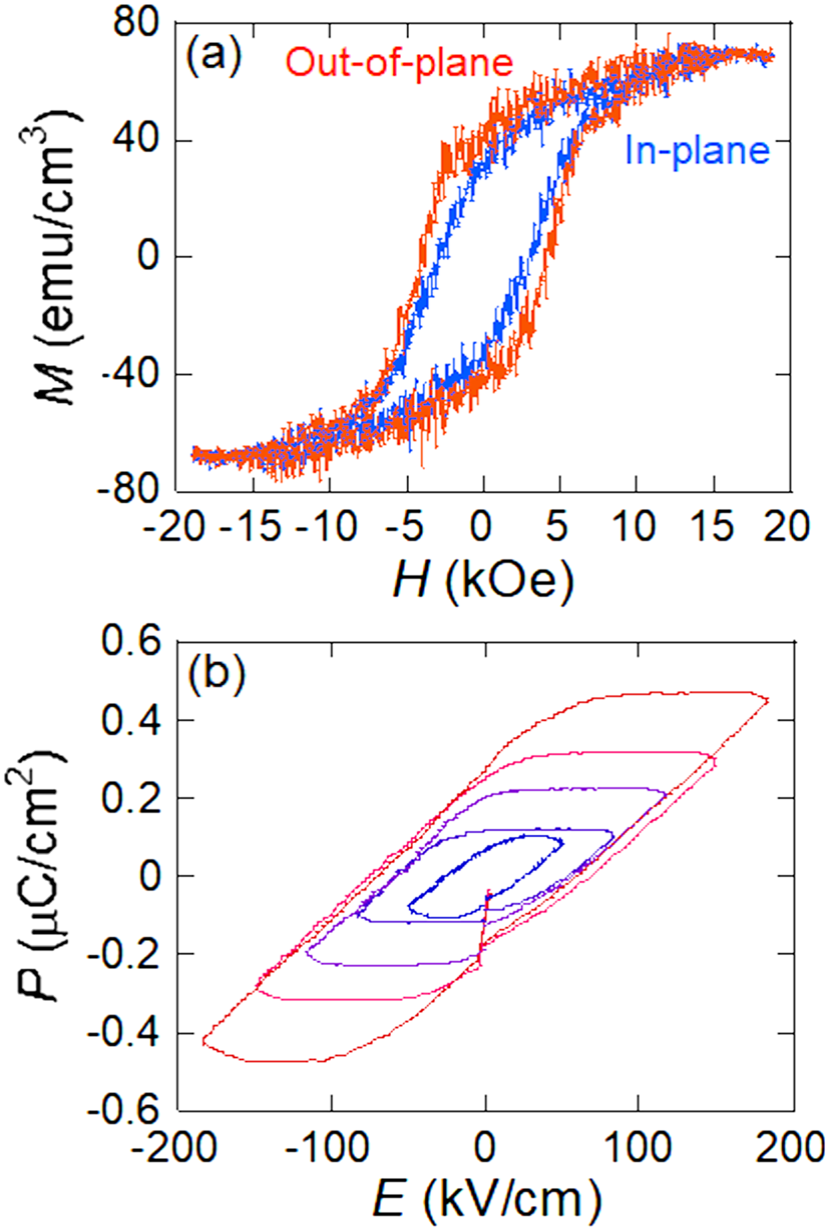
Magnetization curve **(a)** and Ferroelectric **(b)** curves of (Bi_0.41_La_0.59_)(Fe_0.75_Co_0.25_)O_3_ film fabricated by pulsed DC reactive sputtering.

Figure 3 shows the in-plane and out-of-plane magnetization (*M–H*) curves (a) measured by VSM with the magnetic field of parallel and perpendicular to film plane and ferroelectric (*P–E*) curves (b) measured by ferroelectric tester with the electric field perpendicular to film plane of the (Bi_0.41_La_0.59_)(Fe_0.75_Co_0.25_)O_3_ film fabricated by the pulsed DC reactive sputtering. A clear hysteresis loop in both magnetization and ferroelectric curves was observed. The *H*_c_ and squareness (*S*) (= remanent magnetization *M*_r_/*M*_s_) with the in-plane direction of the film were 2.7 kOe and 0.50, respectively. On the other hand, the *H*_c_ and *S* with the out-of-plane direction of the film were 4.2 kOe and 0.64, respectively. This indicates that this film has multiferroic property with ferromagnetism and ferroelectricity. Here, the relationship between the magnetic and ferroelectric properties will be discussed in a future study because only the minor loop of the electric property was measured here. To know the magnetic anisotropy of the (Bi_1−x_La_x_)(Fe_0.75_Co_0.25_)O_3_ film, various *M*-*H* curves were measured by VSM.

**Figure 4:**
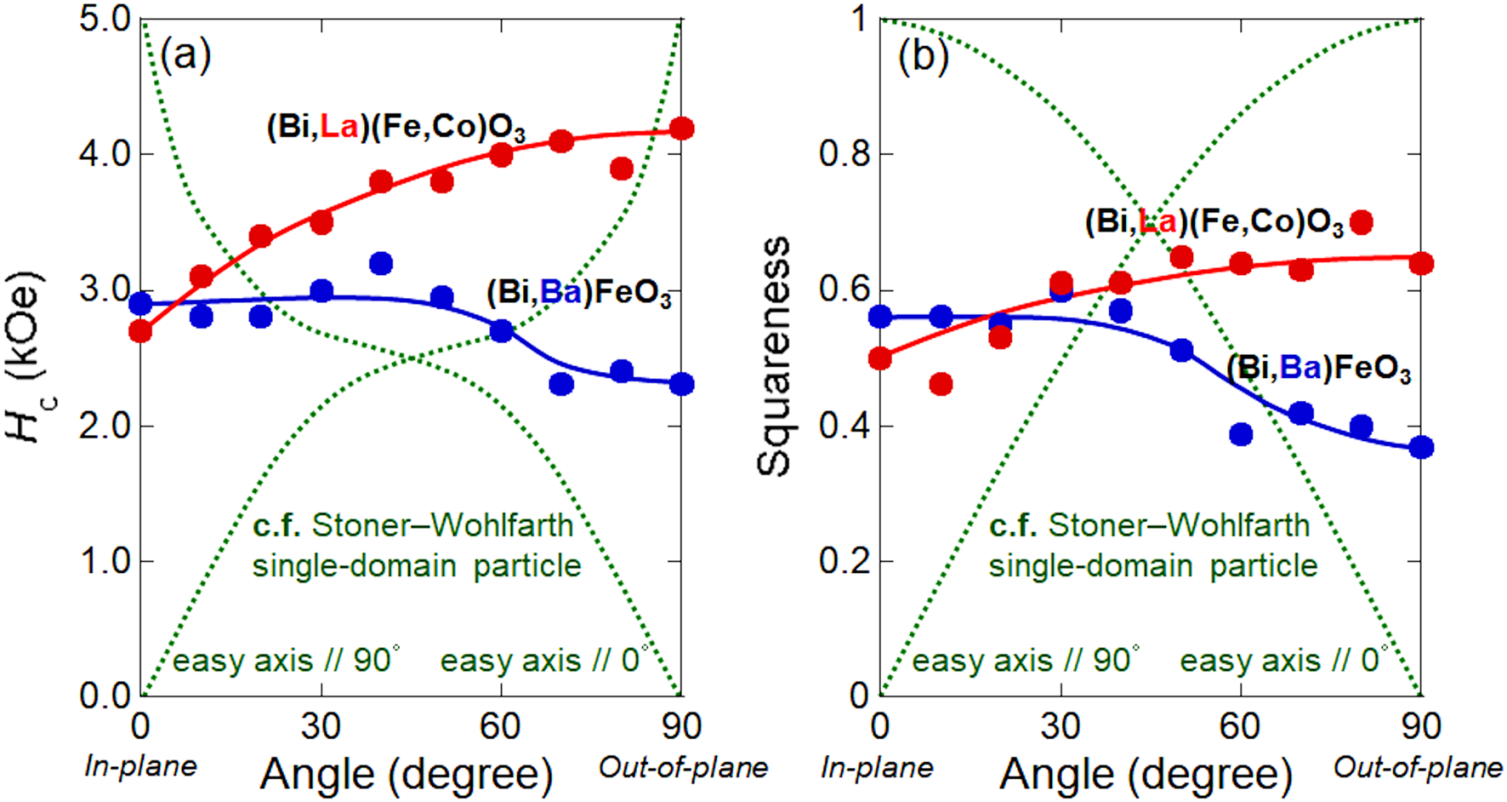
Dependence of coercivity **(a)** and squareness **(b)** on the VSM measuring angle between magnetic field and film.

Figure 4 shows the dependence of the *H*_c_ (a) and *S* (b) on the VSM measuring angle between magnetic field and film plane of (Bi_0.41_La_0.59_)(Fe_0.75_Co_0.25_)O_3_ sample. The *H*_c_ and *S* measured with 0 degree indicates the in-plane *H*_c_ and *S*, and those with 90 degree indicates the out-of-plane *H*_c_ and *S*. The dependence of the *H*_c_ and *S* on the VSM measuring angle between magnetic field and film plane of (Bi_0.48_Ba_0.52_)FeO_3_ film^17^ are also shown in these figures. To recognize the magnetic anisotropy of these films, the dependence of the *H*_c_ and *S* of Stoner–Wohlfarth single-domain particle on the angle between magnetic field and easy or hard axis are also shown in these figures. With increasing the angle, the *H*_c_ and *S* decreased and lowest *H*_c_ and *S* were obtained at 90 degree for (Bi_0.48_Ba_0.52_)FeO_3_ film. This tendency is similar to the case of Stoner–Wohlfarth single-domain particle with the easy axis along 0 degree. On the other hand, with increasing the angle, the *H*_c_ and *S* increased and highest *H*_c_ and *S* were obtained at 90 degree for (Bi_0.41_La_0.59_)(Fe_0.75_Co_0.25_)O_3_ film. This tendency is also similar to the case of Stoner–Wohlfarth single-domain particle with the easy axis along 90 degree. This indicate that (Bi_0.41_La_0.59_)(Fe_0.75_Co_0.25_)O_3_ film has somewhat perpendicular magnetic anisotropy. These magnetic properties are suitable candidate for novel magnetic devices. Here, in our current data about dependence of *M*_s_ and *H*_c_(out-of-plane)/*H*_c_(in-plane) ratio for (Bi,La)(Fe,Co)O_3_ film on Co substitution against Fe, with increasing the Co substitution, both of the *M*_s_ and *H*_c_ ratio increase. However, in the case of (Bi,Ba)(Fe,Co)O_3_ film, both of the (high) *M*_s_ and (low) *H*_c_ ratio do not change against the Co substitution. Therefore, one of the causes of high magnetization and perpendicular magnetic anisotropy for (Bi_0.41_La_0.59_)(Fe_0.75_Co_0.25_)O_3_ film is both substitution of La and Co against Bi and Fe. The details about these mechanisms will be discussed in a forthcoming paper.

**Figure 5:**
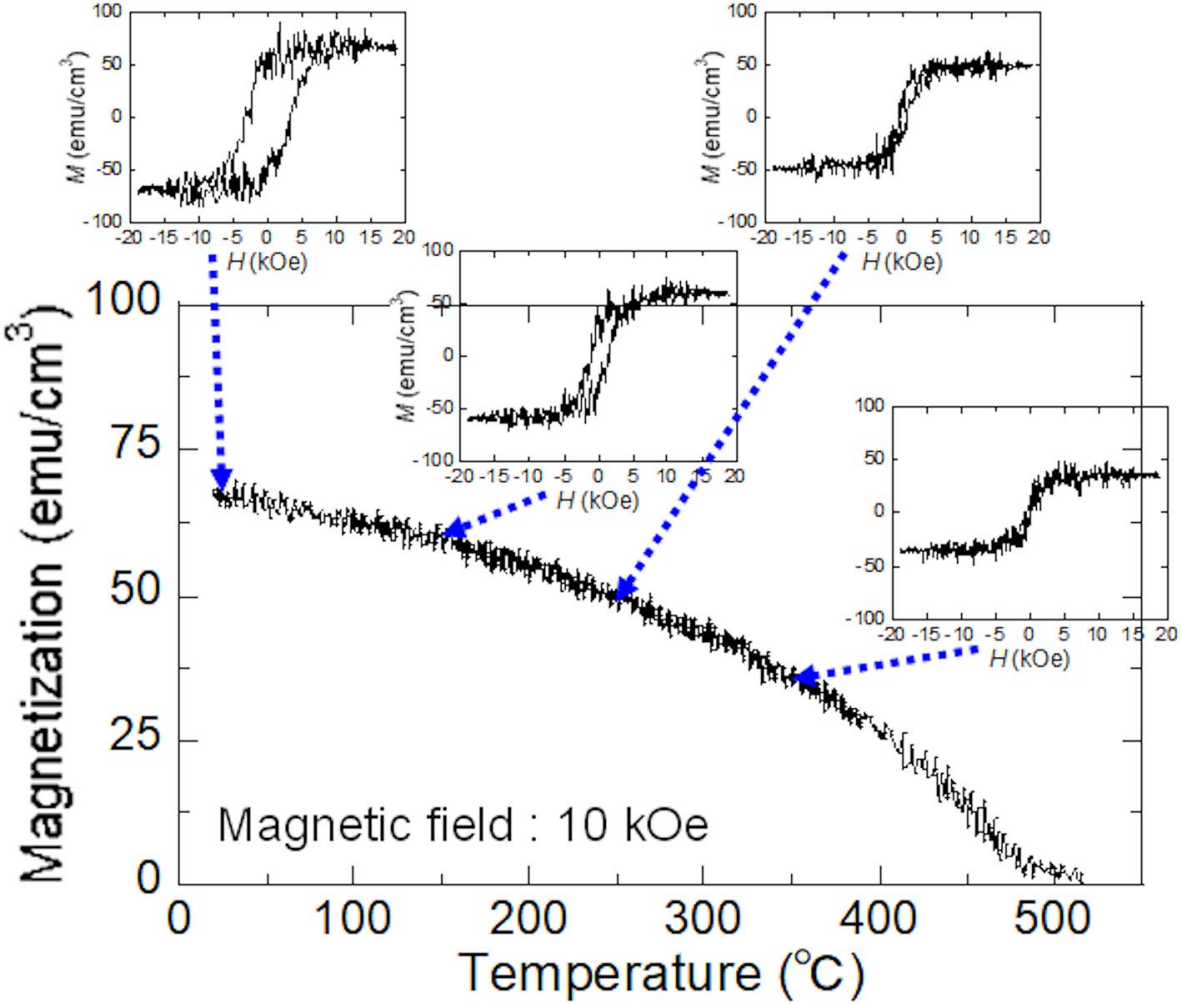
Dependence of saturation magnetization on measuring temperature and the magnetization curve at the measuring temperature of room temperature, 150 °C, 250 °C, and 350 °C for (Bi_0.41_La_0.59_)(Fe_0.75_Co_0.25_)O_3_ film.

Figure 5 shows the temperature dependence of *M*_s_ and the magnetization curve obtained at a temperature of room temperature, 150 ℃, 250 ℃, and 350 ℃ for (Bi_0.41_La_0.59_)(Fe_0.75_Co_0.25_)O_3_ film. A clear hysteresis was observed and *T*_C_ was approximately 420 ℃, which was estimated from d*M*/d*T* plot. This property is also useful for application to practical magnetic devices.

**Figure 6:**
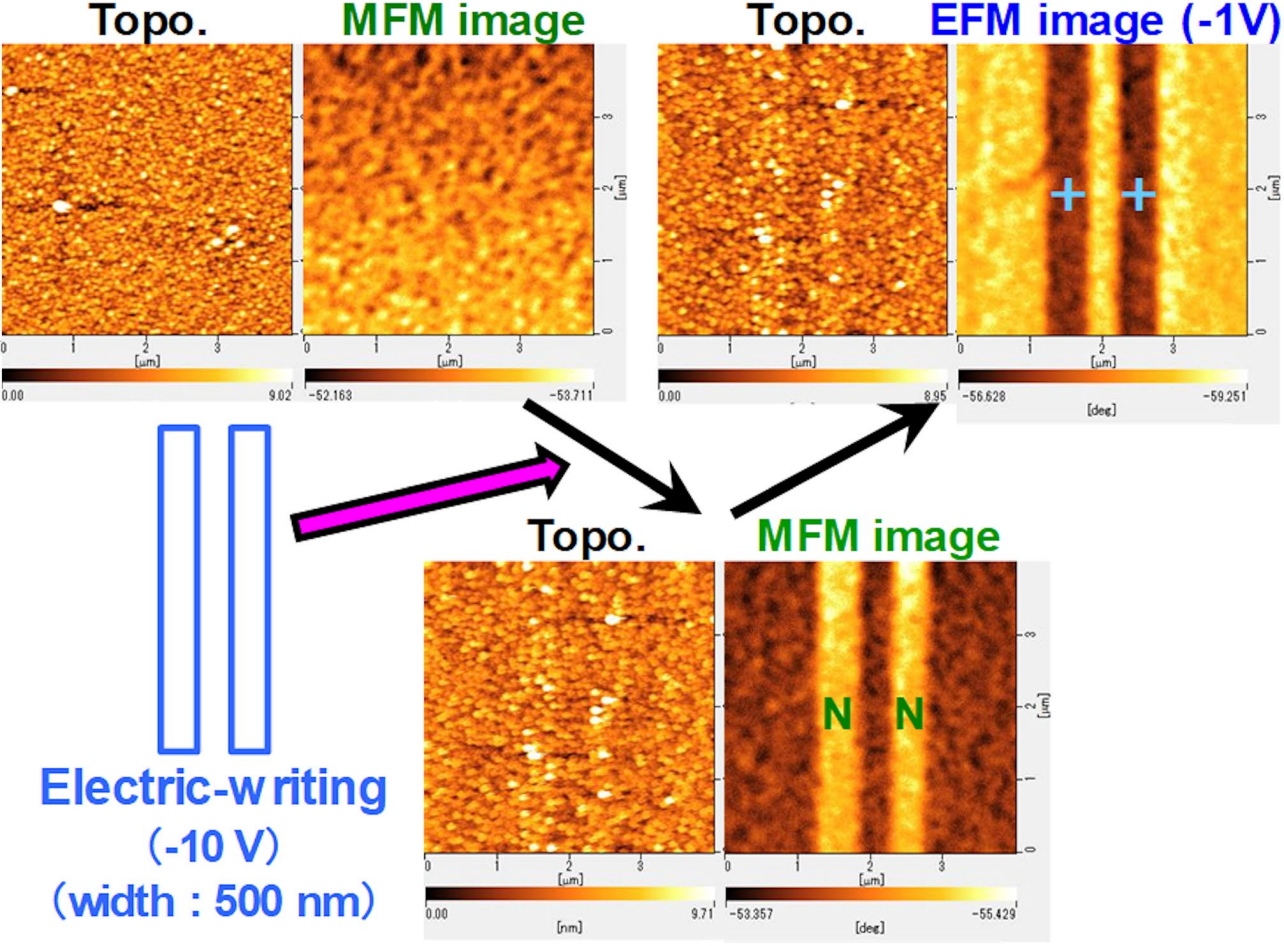
Topographic, MFM (tip end: N), and EFM (tip end: –) images of (Bi_0.41_La_0.59_)(Fe_0.75_Co_0.25_)O_3_ thin film of before and after applying DC voltage of − 10 V.

Figure 6 shows topographic (measured by atomic force microscope (AFM)), magnetic (measured by MFM), and electric (measured by EFM) images of (Bi_0.41_La_0.59_)(Fe_0.75_Co_0.25_)O_3_ film of before and after local electric field writing by conductive tip with − 10 V. The scan parameters for the writing process are “contact mode” with the scan speed of 5 µm/s and steps of every 10 nm. In previous study, the spatial resolution of our MFM measurement was around 10 nm^22^, which is sufficient for MFM measurement of magnetic domains with several hundred nano-meter width. The MFM image of (Bi_0.41_La_0.59_)(Fe_0.75_Co_0.25_)O_3_ film of before local electric field writing showed de-magnetized state. However, the MFM image of (Bi_0.41_La_0.59_)(Fe_0.75_Co_0.25_)O_3_ film after local electric field writing showed clear magnetic domain which magnetization direction is from down to up. This contrast pattern of MFM image is similar to EFM image. The reason of different of contrast color is that the applied low voltage to Co–Cr–Pt tip for EFM measurement was − 1 V. If we will choose the applied low voltage of + 1 V to tip for EFM measurement, the contrast color of EFM image is similar to the color of MFM image. We used low voltage of − 1 V to tip for EFM measurement to distinguish two images between MFM and EFM. This indicates that magnetization with the width of 500 nm is induced by the local electric field writing.

**Figure 7:**
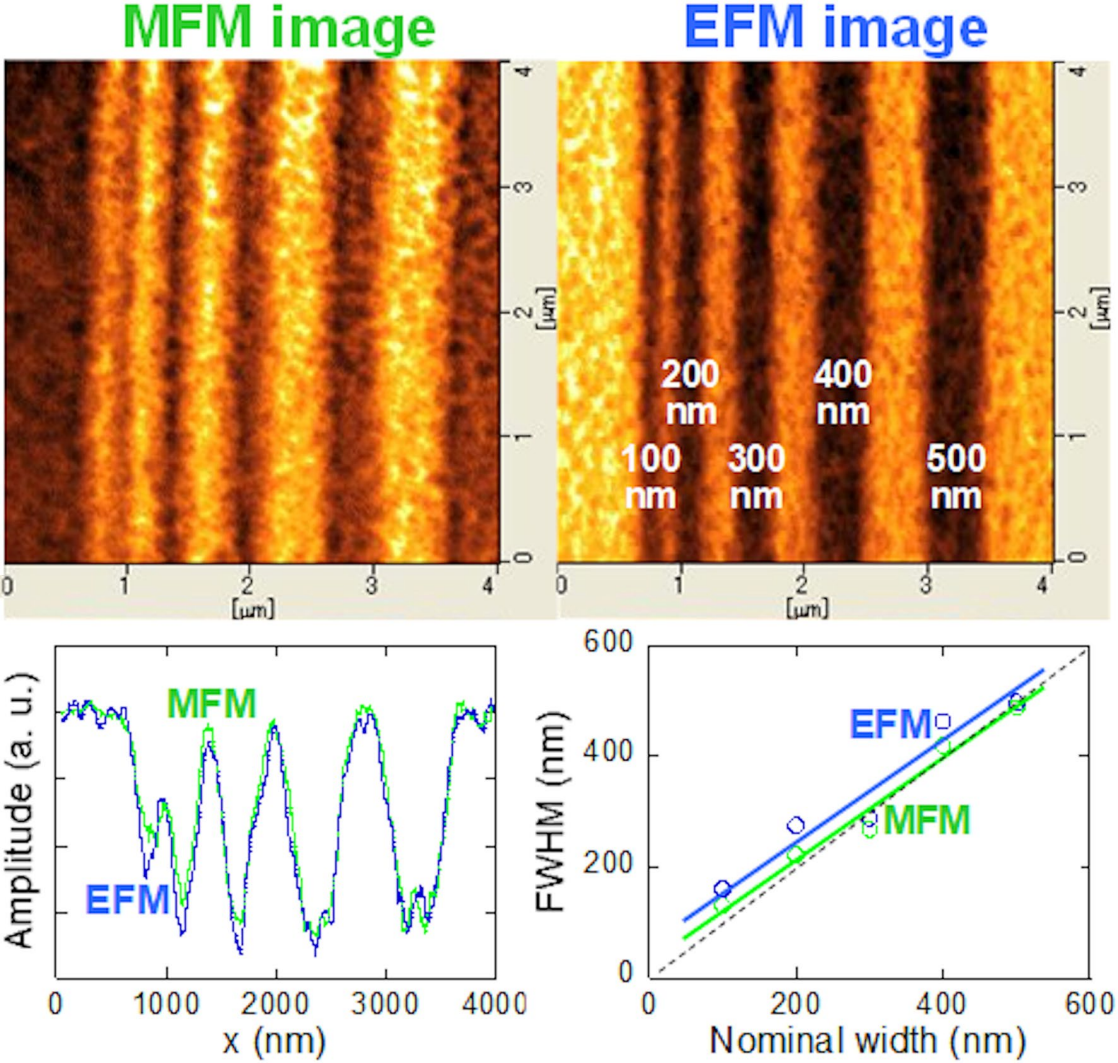
MFM (tip end : N), and EFM (tip end :–) images of (Bi_0.41_La_0.59_)(Fe_0.75_Co_0.25_)O_3_ thin film after applying DC voltage of − 10 V with various widths. The line profile of both images and full width at half maximum of each domain.

We examined how thin can be written in this (Bi_0.41_La_0.59_)(Fe_0.75_Co_0.25_)O_3_ film. The width of local electric field writing by conductive tip with − 10 V was varied from 500, 400, 300, 200, to 100 nm. The MFM (tip end: N), and EFM (tip end: –) images of (Bi_0.41_La_0.59_)(Fe_0.75_Co_0.25_)O_3_ film after applying DC voltage of − 10 V with various widths are shown in Fig. 7.

The line profiles of both images and full width at half maximum (FWHM) of each line profiles of induced domains with various widths are also shown. In the case of the line profile of MFM, peaks and valleys are reversed for easy comparing between line profiles of MFM and EFM. Down to the 300 nm width, the FWHM of each domains was similar to nominal value of electric field writing. However, less than 300 nm width, the FWHM of each domains was larger than nominal value of electric field writing. From these results, it is found that an electric field writing of down to 300 nm width, which is a level close to the width of the magnetization reversal region expected in magnetic device applications, is possible. Here, the reason of different FWHM between MFM image and EFM image should be discussed. The domain boundary of MFM image is not sharp compared with EFM image. This can be understood from line profile of especially narrow domains such as 100, 200 nm width. The slope of line profile from peak to valley and width of valley bottom in the case of MFM image are smaller than the case of EFM image. Generally, minimum domain width is several hundred nano-meter in ferromagnetic films and minimum domain width is several tens nano-meter in ferroelectric films. The reason is that the interaction between magnetic moments is very large compared with the case of electric moments. Therefore, this is reason about that the domain boundary of MFM image is not sharp compared with EFM image. On the other hand, minimum domain width of the (Bi,La)(Fe,Co)O_3_ film is around 100 nm, which is intermediate domain width between ferromagnetic and ferroelectric films. This indicates that the interaction between magnetic moment and electric moments exists. Based on the above, we have succeeded in demonstrating magnetization reversal on the submicron scale by applying a local electric field. This technique will be useful for realization of new magnetic devices.

## Methods

### Film fabrication

Multilayers of Ta (5 nm)/Pt (100 nm) / (Bi_1−x_La_x_)(Fe_0.75_Co_0.25_)O_3_ (300 nm) were deposited onto a thermally oxidized Si wafer using a UHV sputtering system (Eiko, ES-360-AK). The La concentration x was varied from 0.44 to 0.68. The Ta seedlayer, Pt underlayer, and (Bi_1−x_La_x_)(Fe_0.75_Co_0.25_)O_3_ layer were deposited at room temperature, 300 °C, and 570 °C, respectively. The film thickness and deposition temperature of the Ta seedlayer and Pt underlayer were optimized to obtain a strong (111) orientation of the Pt underlayer^20^. The very high frequency (VHF) (40.68 MHz) plasma irradiation^21^ during the reactive pulsed DC sputtering deposition of (Bi_1−x_La_x_)(Fe_0.75_Co_0.25_)O_3_ films was performed with an electric power of 5 W to obtain the crystal grain growth of (Bi_1−x_La_x_)(Fe_0.75_Co_0.25_)O_3_ thin films. The frequency of pulsed DC was fixed with 200 kHz. Here, the duty ratios between sputtering ON and OFF are 3 and 2, for example, the time of sputtering ON is 3 µs and that of OFF is 2 µs for this condition. The sputtering power of pulsed DC was fixed with 150 W. The details of pulsed DC sputtering source (ULVAC, DPG-P5) are described in the reference numbers 18 in the reference list. The details of fabrication of BiFeO_3_-based thin films by using a pulsed DC reactive sputtering method are described in the reference numbers 17 in the reference list.

### Measurement

The composition of the fabricated (Bi_1−x_La_x_)(Fe_0.75_Co_0.25_)O_3_ films was analyzed by energy dispersive X-ray spectroscopy (EDS) (JEOL, JSM-5900LV). The crystallographic orientations and crystalline structures of the fabricated (Bi_1−x_La_x_)(Fe_0.75_Co_0.25_)O_3_ films were analyzed by X-ray diffraction (XRD) analysis (BRUKER, D8 ADVANCE). The magnetization curves of (Bi_1−x_La_x_)(Fe_0.75_Co_0.25_)O_3_ were measured using a vibrating sample magnetometer (VSM) (Toei, VSM-5S) with the application of a magnetic field of in-plane direction, out-of-plane direction, and various angle to the film surface. The ferroelectric hysteresis loops of the (Bi_1−x_La_x_)(Fe_0.75_Co_0.25_)O_3_ films were measured using a ferroelectric tester (TOYO, FCE-1E). The local electric field writing, and the electric and magnetic domain analyze were performed by scanning force microscopy (SPM) (SII, SPI-3800). The local electric field was applied to the (Bi_1−x_La_x_)(Fe_0.75_Co_0.25_)O_3_ film using an atomic force microscopy (AFM) of “contact mode” with a conductive, magnetic Co-Cr-Pt tip. The electric and magnetic domain structures of the (Bi_1−x_La_x_)(Fe_0.75_Co_0.25_)O_3_ film were analyzed by electric force microscopy (EFM) and magnetic force microscopy (MFM), respectively, with a conductive, magnetic Co-Cr-Pt tip (Manufacturer: Hitachi High-Tech Science Corporation, Model number: SI-MF40-Hc).

## Conclusions

In summary, we fabricated high quality multiferroic (Bi_1−x_La_x_)(Fe,Co)O_3_ magnetic films using pulsed DC reactive sputtering technique. The magnetization (*M–H*) curve of out-of-plane and ferroelectric (*P–E*) curve of (Bi_1−x_La_x_)(Fe,Co)O_3_ shows clear hysteresis. The squareness (*S*) and coercivity (*H*_c_) of perpendicular to film plane are 0.64 and 4.2 kOe which are larger compared with films in parallel to film plane 0.5 and 2.5 kOe. Curie temperature (*T*_C_) of (Bi_1−x_La_x_)(Fe,Co)O_3_ is 420 ℃, which is clearly higher than room temperature. The submicron-scale magnetization was introduced by applying a local electric field and that the directions of polarization and magnetization are parallel. This indicates the proposed multiferroic (Bi_1−x_La_x_)(Fe,Co)O_3_ films are expected to be useful in novel magnetic devices driven by electric field.
